# Supplementary material for: Functional Identification of HhUGT74AG11—A Key Glycosyltransferase Involved in Biosynthesis of Oleanane-Type Saponins in Hedera helix
Source: Int J Mol Sci. 2024 Apr 5;25(7):4067. doi: 10.3390/ijms25074067 (PMC11012674; doi:10.3390/ijms25074067)
Supplement: Supplementary file 1 [file ijms-25-04067-s001.zip › ijms-2906835-supplementary.pdf]

Supplementary Material

Supplementary Figures

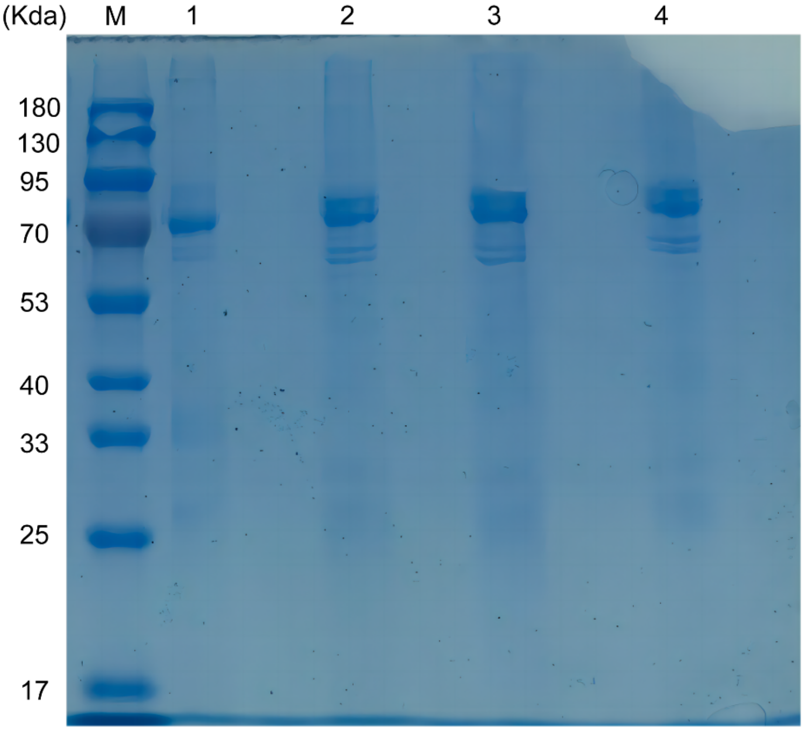

Supplementary Figures S1. SDS-PAGE of recombinant *HhUGTs* purified using GST affinity chromatography. M, standard protein markers (PageRuler™ Prestained Protein Ladder, 10-180 kDa, Thermo). 1, Unigene26859 (*HhUGT74AG11*); 2, Unigene31717; 3, CL11391.Contig2; 4, CL144.Contig9.

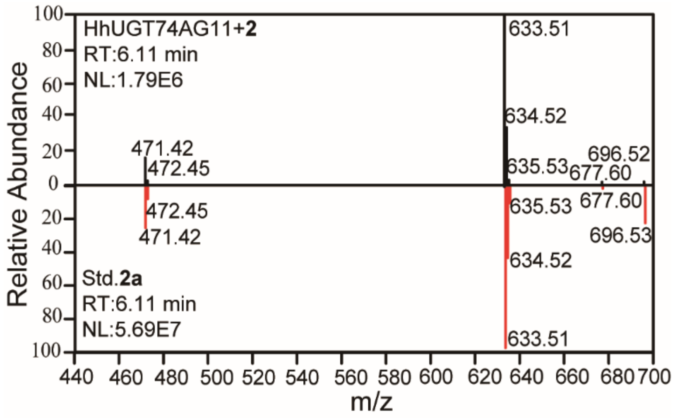

Supplementary Figures S2. Hederagenin (2) as the substrate of *HhUGT74AG11*. MS/MS fragmentation of the product peaks by *HhUGT74AG11* + **2** compared with fragmentation results of the Std. **2a** ([(-)-mode], m/z = 633.51).

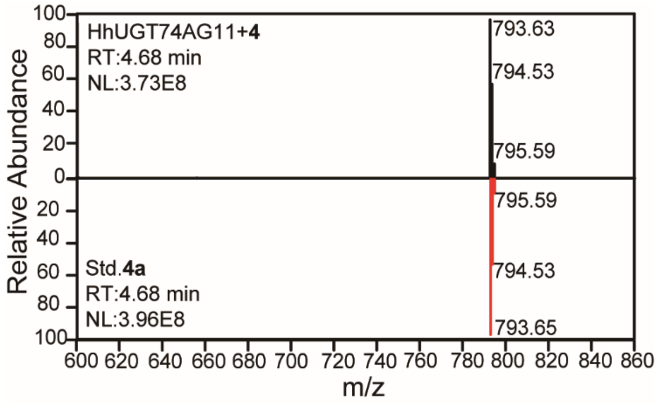

Supplementary Figures S3. Calenduloside E (4) as the substrate of *HhUGT74AG11*. MS/MS fragmentation of the product peaks by *HhUGT74AG11* + **4** compared with fragmentation results of the Std. **4a** ([(-)-mode], m/z = 793.65).

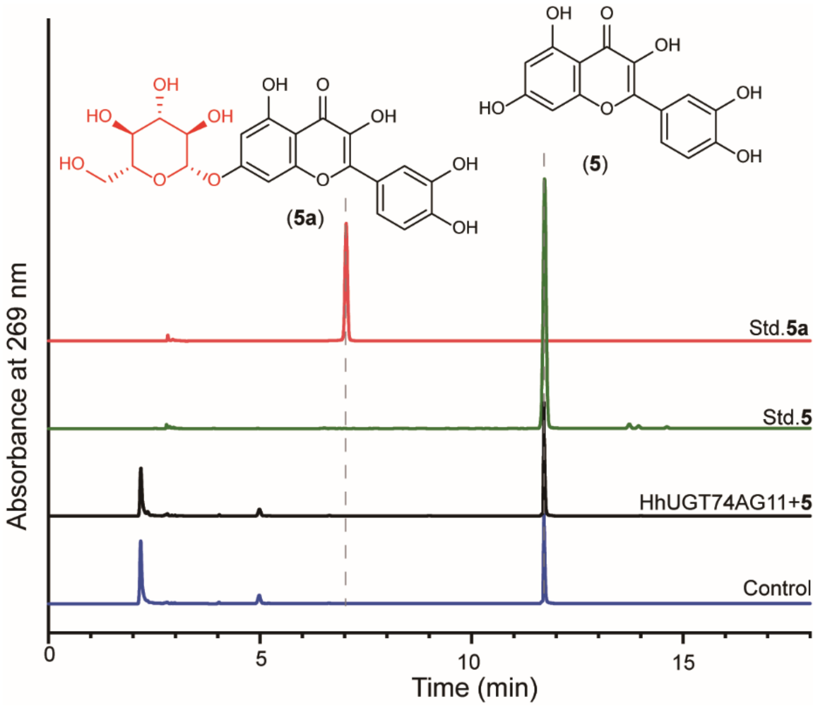

Supplementary Figures S4. Quercetin 5 as the substrate of *HhUGT74AG11*. HPLC analysis (monitored at 269 nm) of the reaction mixtures; quercetin-7-*O*-β-D-glucopyranoside **5a**, quercetin **5**.

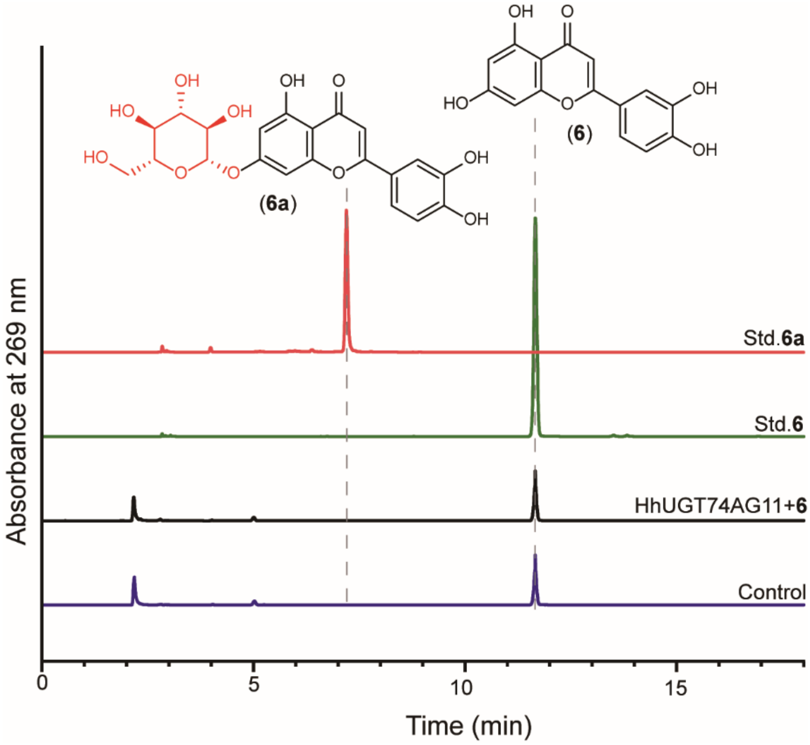

Supplementary Figures S5. Luteolin 6 as the substrate of *HhUGT74AG11*. HPLC analysis (monitored at 269 nm) of the reaction mixtures; cynaroside **6a**, luteolin **6**.

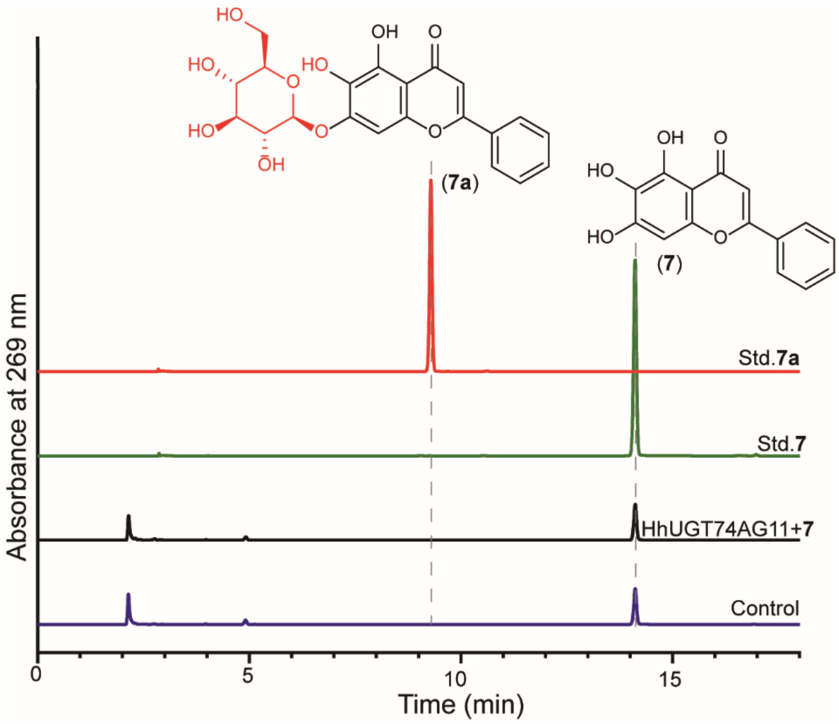

Supplementary Figures S6. Baicalein 7 as the substrate of *HhUGT74AG11*. HPLC analysis (monitored at 269 nm) of the reaction mixtures; oroxin A **7a**, baicalein **7**.

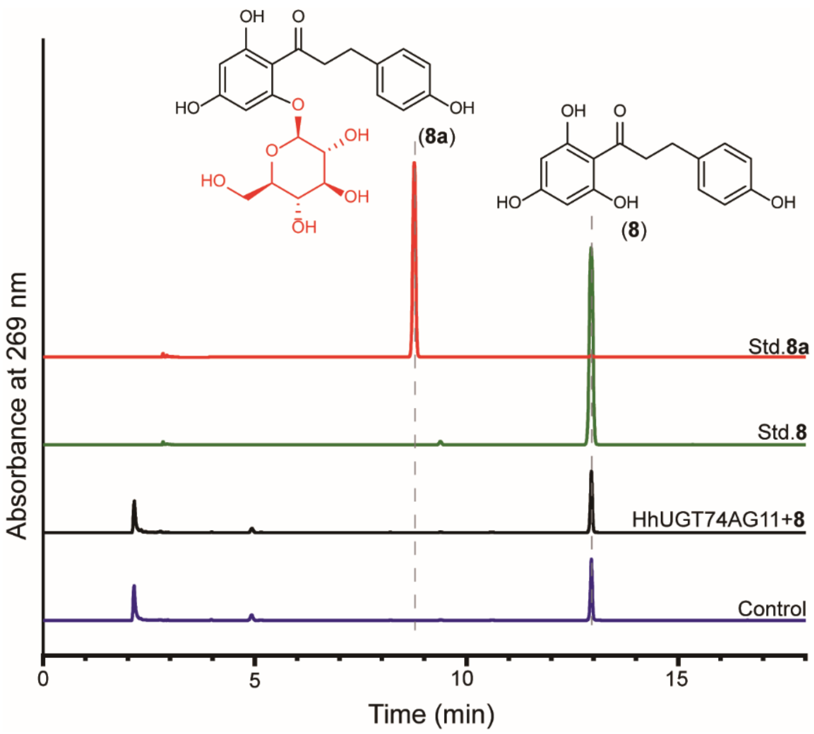

Supplementary Figures S7. Phloretin 8 as the substrate of *HhUGT74AG11*. HPLC analysis (monitored at 269 nm) of the reaction mixtures; phlorizin **8a**, phloretin **8**.

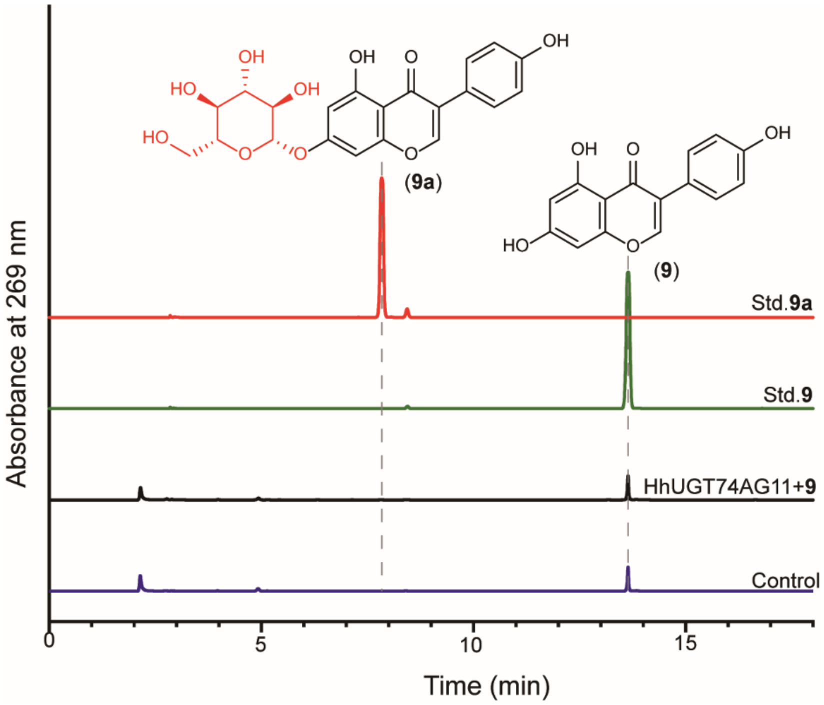

Supplementary Figures S8. Genistein 9 as the substrate of *HhUGT74AG11*. HPLC analysis (monitored at 269 nm) of the reaction mixtures; genistin **9a**, genistein **9**.

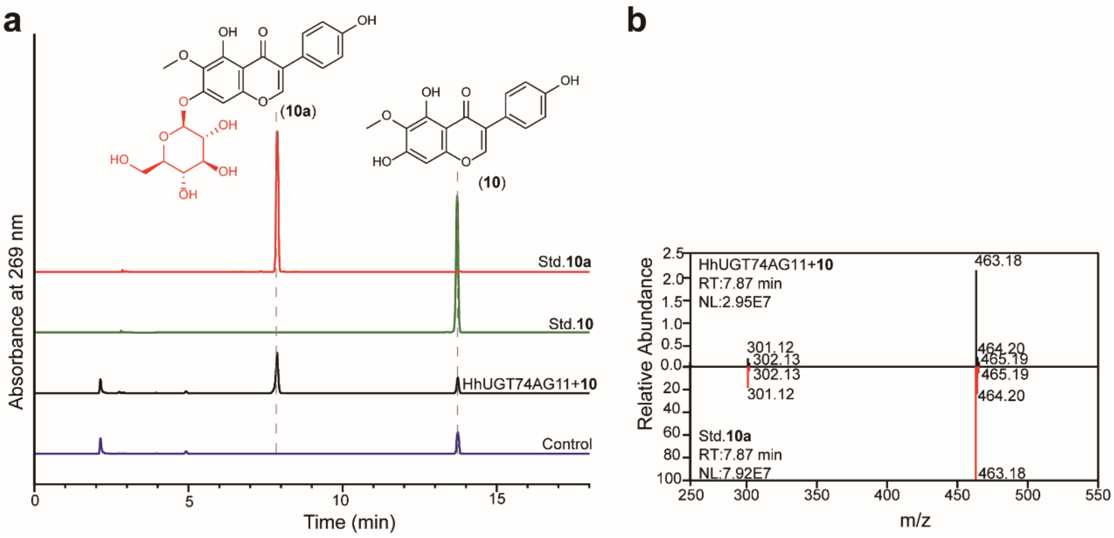

Supplementary Figures S9. Tectorigenin 10 as the substrate of *HhUGT74AG11*. a: HPLC analysis (monitored at 269 nm) of the reaction mixtures; tectoridin **10a**, tectorigenin **10**; b: MS/MS fragmentation of the product peaks by *HhUGT74AG11* + **10** compared with fragmentation results of the Std. **10a** ([(-)-mode], m/z = 463.18).

Supplementary Tables

Table S1. Primers used in this study.

Primer	Sequences (5′-3′)
Unigene26859 (*HhUGT74AG11*)-Forward primer	gatctggttccgcgtggatccATGGAGAATGAGAAAACTTATAAAGCTC
Unigene26859 (*HhUGT74AG11*)-Reverse primer	ctcgagtcgacccgggaattcTTAGAGTGCCAAAATCCGAGAAA
Unigene31717-Forward primer	gatctggttccgcgtggatccATGGCCGCGAATGACAAA
Unigene31717-Reverse primer	ctcgagtcgacccgggaattcTCATTTTTTGAGGATTTTATGATTTTC
CL11391.Contig2-Forward primer	gatctggttccgcgtggatccATGGCGGTGGCCGGCGCC
CL11391.Contig2-Reverse primer	ctcgagtcgacccgggaattcTTAGTATTCAGATAAATGTCTAACCAACTCA
CL144.Contig9-Forward primer	gatctggttccgcgtggatccATGGAAGAGAGAAAAGGAAAGACCA
CL144.Contig9-Reverse primer	ctcgagtcgacccgggaattcCTATTCATTCTCCCCTCTTGGCT

Table S2. The expression profiles of 82 unigenes in this study.

geneID	L1	L2	L3	R1	R2_	R3
CL10734.Contig3	0.9915	0.8409	1.4965	0.6242	0.3793	0.6069
CL8643.Contig2	0.7677	0.9567	0.713	1.0494	1.9018	0.8593
Unigene7122	0.3531	0.3423	0.3423	0.3423	0.7204	3.8043
CL4883.Contig1	2.5633	1.5719	2.9153	5.0056	1.8678	8.249
CL5071.Contig1	1.6293	2.969	2.1792	3.7417	3.1247	4.3405
CL84.Contig7	1.2457	1.2074	1.3612	7.7864	3.1021	7.0639
Unigene12948	0.2697	0.6151	0.6189	0.3356	0.1456	0.4506
CL10135.Contig5	1.6004	1.7936	1.7652	5.0878	4.1325	5.4862
Unigene31942	1.6249	1.1812	1.0061	0.2728	0.5525	0.6631
CL2755.Contig4	0.0381	0.0739	0.1062	0.0384	0.2332	0.4851
CL1653.Contig1	3.5483	4.0427	2.7175	4.0755	4.8897	6.8895
CL2011.Contig4	14.5591	18.1027	16.3217	22.1462	21.1738	19.7054
Unigene1574	3.6483	2.5718	0.9242	2.3384	0.3383	0.6497
Unigene19763	0.8496	1.3528	0.9018	2.3226	2.1666	1.4858
Unigene19769	0.671	1.0643	0.7932	1.1059	1.1201	1.0754
Unigene24545	0.7998	0.5814	0.7163	3.136	2.3894	4.4202
CL1679.Contig2	1.8171	2.418	1.1156	1.8922	1.885	2.2019
CL1680.Contig1	1.8354	2.1906	2.0482	6.1806	6.0641	3.7696
CL1710.Contig3	0.2427	0.2521	0.1288	5.3612	6.4556	4.8566
CL1719.Contig2	5.7806	3.9834	3.1879	4.7762	8.1082	5.0866
CL1741.Contig3	67.6352	74.3865	94.6205	10.4303	11.4763	6.3986
CL6964.Contig4	0.3123	0.2018	0.1934	1.1533	2.4423	7.7483
CL7506.Contig1	3.1544	4.3933	3.3565	3.9792	4.3425	3.6593
CL4453.Contig5	2.5397	2.8625	2.4686	2.8556	4.6394	3.4709
Unigene20397	4.5627	5.7292	5.2974	2.3501	3.5967	5.0782
Unigene27513	0	0.2729	0.3922	3.2609	2.7282	1.6543
Unigene37653	1.6776	1.084	0.2597	0.5632	1.9965	0
CL12103.Contig1	1.1888	1.8536	1.3922	1.4056	1.8981	1.873
CL3433.Contig7	1.7506	2.4824	1.5055	3.102	3.4393	1.4288
CL718.Contig1	16.536	11.3226	17.0027	29.7457	23.2137	19.6131
Unigene32600	0.6183	3.2961	1.7228	0.3114	0.3154	0.3028
CL12227.Contig3	46.0956	53.1297	39.9672	4.7912	7.5326	4.2691
CL308.Contig1	7.0386	8.149	9.7154	7.0895	5.1857	8.7129
Unigene21454	4.0585	1.7484	0.8377	0.1514	0.1533	0.2944
Unigene22346	1.1258	1.0912	0.8043	2.0934	1.1042	1.4843
CL3923.Contig4	61.2911	64.444	76.5068	19.4209	25.1727	22.3582
CL8585.Contig2	2.1114	1.3449	2.0731	0.1052	0.1052	0.1052
Unigene7386	2.8003	4.1405	4.2762	3.6811	2.082	4.8811
CL6504.Contig2	92.4307	57.1962	114.7202	0.5237	0.5304	0.4365
Unigene15662	25.0961	14.7295	26.5278	9.772	5.1505	12.5565

Unigene7507	31.3605	27.2478	33.8378	11.5539	4.3693	9.3544
CL6504.Contig2	92.4307	57.1962	114.7202	0.5237	0.5304	0.4365
CL9981.Contig2	45.8707	39.9531	36.7244	0.5206	0.1318	0.5062
CL9981.Contig1	7.2625	5.2092	5.5313	0.0001	0.0001	0.0001
CL5897.Contig1	43.4022	14.2551	42.7668	53.7554	3.9851	89.6598
CL720.Contig1	0.3635	0.2883	0.2455	1.2315	0.6405	0.2589
Unigene17579	14.1423	12.9285	18.4512	3.8113	10.229	9.0334
CL9843.Contig1	3.4026	2.9212	3.5215	5.0265	3.5372	5.7447
CL8670.Contig1	429.2733	336.8246	574.4803	132.8436	14.1181	196.9498
CL2359.Contig2	75.6548	48.5475	174.78	0.0921	0.0467	0.1344
Unigene20154	0.7104	0.4734	0.0825	7.2451	10.1008	9.4804
Unigene8931	2.3476	1.6118	2.3622	11.1332	17.1629	7.4248
Unigene10909	53.3615	46.9282	79.2753	38.0871	36.9196	40.5264
CL2288.Contig1	130.9246	92.8239	146.6269	0.2656	0.0897	0.2583
Unigene26859	4.4025	4.0163	3.4477	14.1725	16.5554	28.1965
Unigene6593	31.1151	5.8765	52.6342	0.6056	0.0511	1.1287
Unigene22454	35.6495	19.5374	76.1373	6.4195	1.0572	3.8062
Unigene31717	0.2961	1.148	0.4584	109.6537	240.5387	108.3176
CL7205.Contig1	63.1729	56.0285	63.0994	0.5233	0.0883	0.1696
CL9425.Contig1	29.7613	15.6297	57.404	0.4178	0.2116	0.6094
Unigene27341	12.9111	9.5695	22.1988	0.4661	1.8072	0.92
CL8278.Contig2	120.4899	414.6659	376.3691	5.2658	4.0753	15.7959
CL8684.Contig2	26.2099	36.7485	23.2495	1.9646	0.2902	1.1542
CL4177.Contig2	122.1633	171.6265	180.4338	77.061	58.0098	56.7277
CL6439.Contig1	5.8063	23.1769	20.7382	11.6965	6.8385	7.703
CL11391.Contig2	25.4757	21.0486	20.7154	65.2426	79.1428	76.6022
Unigene13588	17.586	18.561	9.8458	2.4602	4.4351	1.0526
CL12077.Contig3	62.3858	52.4807	79.0922	16.8788	19.2316	25.1098
CL8156.Contig9	22.7943	17.6307	27.7155	17.6252	16.1596	24.941
CL11669.Contig2	21.2336	31.9446	28.6428	7.0658	5.3792	9.7758
CL419.Contig1	53.4301	52.3786	65.4227	2.2056	1.936	0.9294
CL10096.Contig2	92.8695	63.4411	56.459	0.4315	0.7284	0.4662
CL10480.Contig1	454.6486	348.7123	575.5306	1.2092	0.2449	1.2698
Unigene20624	10.6391	11.6574	12.3736	8.2933	8.4666	8.8345
Unigene23490	27.6322	104.0617	91.7902	0.1228	0	0.0398
CL144.Contig9	3.1617	3.9337	2.7613	5.371	8.5687	3.3739
Unigene25019	68.8753	57.6478	75.8665	6.0388	7.5349	17.8509
CL10438.Contig2	33.0103	30.2388	37.6128	26.4587	30.1172	22.7682
CL1235.Contig3	15.3708	27.4234	44.9475	2.5094	2.7569	4.8389
Unigene27253	9.8195	10.5903	12.4603	0.9751	0.4703	0.6321
CL11671.Contig1	95.1753	74.2572	70.5888	42.6534	32.2674	33.2357

Table S3. Amino acid sequences of *HhUGTs* candidates.

> Unigene26859 (*HhUGT74AG11*, OR902350)

MENEKTYKAHIMVLAYHGQGHINPMVQFSKRLASKGMKITVTTTLSNTKAVKTASSS
VILESIYDDATEGGVGAPGGFKGFLDRFEASGSRNLIELIKKQENSGYPIKCLVYDANIP
WASNIAKQFAIPGAAFFTQSCAAVASYYPMHCDLSEKSLPFPAFSMPGLPPPKLLYLPS
LGAVTGQYSPIIRYICKQFDNIENAEWVLFNSFDKLEEEVVKWMSNLWTVRNIGPTVP
SVYLDNRVENDSDYGFNLFKPSTEVCMQWLNTKETGSVVYVSFGSAASLSAEQMAEM
AEALKQSRNSFLWVVKPTEESKLPTNFIEETSEKGLVVTWCPQLEVLAHHAVGCFISH
CGWNSTVEAISFGVPVVAMPQFLDQMTNAYFVEKVWGVGIQPKENEENVTSAEEIGR
CINEVMDGEEIKKKAMQWKELAKEAIDENGSSDKSIDEIISRILAL*

> Unigene31717

MAANDKLHIVMFPWLAFGHILPYLKLAKLIAKKGHKISFISTPRNIDRLPKIPPNVAPLI
DLVKFPLPSIPNLPENAEATMDVPFNEVKYLKIAYDQLQQPLTQFLESNSPNWILFDLIP
YWVGPIASKLNVRSAFFSIFSASQLGYFGPPSEMMDGDEDHQKPEDYTIKPKWVRFETS
VAPSLHQMQRVFVNFTKDDTENVPDLYRFAASIRDCDMVIIRSSSEFEPQWLELLDEIY
EKPVVQVGLLPTDTTDTDTGNESNDSWRDIKDWLDKQEEGSVIYIAFGAETKPNQDEL
TELALGLELSRLPFIWAIRKQRGLADPEPTELPEGFEERTRGRGLVYTTWVPQTKILSHD
SVGGLLIHSGWSSVIEAVQFGRAMILLPFLGDQELIAKLVEEKKLGYLIPRNERDGWFSR
DSVAESVRLVMVEEEGKIYRTNVKEMKGVFGDMDKQDKYVDNLLSFLENHKILKK*

> CL11391.Contig2

MAVAGAGAHILIFPYPAQGHMIPLLDLTHQLAIRGLTITILVTPKNLSLLNPLLSKHPSIK
PLVLPFPATPSIPDGVENVKDLPVGGFRAMMTALGKLYNPILNWFQSHPSPPVAIISDM
FLGWTHRLACQLSIRRFVFCPSGALAMSVIFALWRDMPQKNDHCDENELISFPEIPNSP
VYPWWQLSTVYRSYIAGDPQSEFLKDSFRGNIASWGLVINSFSELERVYLDYLKESLGH
DRVWPVGPLLPPENDRVSRGGSSLVLDSEITSWLDKFEDRTAVYVCFGSQAVLTNKQM
EELALGLEKSGVTFLWSSKIPTKGHVEGLYGVMPSGFEDRVAGRGLVIKEWAPQVTILS
HRAVGTFLTHCGWNSVLESIVAGVPMLAWPMGADQFTNADLLDEVKVGVRVCEGES
TVPDSDQLARLLAKAVSNEERVETIVTAKELSKAALNSTKMGGSSYKSVDELVRHLSEY
*

> CL144.Contig9

MEERKGKTIVLFPYMAQGHIIPFLALALQIEKKGYQITFVNTPLNIKNLRQSLPLNTSIRL
LEIPFNSSHHRLPPETENTDGVNFSLTLTLLEASVSLKPAFRNLISDLVRCGAQPLAIIAD
IFLGWTAEVAHEFGIFHTIFSSTGGFGMACYYSVWMNLPHNNTDSVEFTLPDFPEAGRI
HRTQLSANVLAADGTDPWSKILQLLLSSWVDSDGILFNTIEEIDKIGLHYFRKKLSLPV
WPIGPILLSVDTRAGTSENPGISSESCINWLDSKPANSVIYVSFGSQNTISASQMMQLAK
ALDSIDINFIWVVRPPLGFDINLEFHAEEWLPEGFLKRIEDQNRGLIIVKWAPQVEILLH
KAVAAFLSHCGWNSLLESLSGGVPLIGWPMGAEQFYNVKYLEEEVGVCVELARGTNF
EVRSEDIVEKIGILMRENGKGKEMREKACEVKKMIEDGGRDEEGYKGSSVKAMEEFLN
VAAKEKKPRGENE*

Table S4. Protein sequences used for phylogenetic analysis with their corresponding accession numbers.

Protein Name	*Plant species*	*Accession No.*
UGT71		
AtUGT71B5	*Arabidopsis thaliana*	OAO99526.1
AtUGT71B6	*Arabidopsis thaliana*	*Q9LSY6.1*
AtUGT71B7	*Arabidopsis thaliana*	Q9LSY5.2
AtUGT71B2	*Arabidopsis thaliana*	Q9LSY8.1
AtUGT71B8	*Arabidopsis thaliana*	Q9LSY4.1
AtUGT71B1	*Arabidopsis thaliana*	Q9LSY9.1
AtUGT71C1	*Arabidopsis thaliana*	O82381.1
AtUGT71C2	*Arabidopsis thaliana*	OAP11738.1
AtUGT71D1	*Arabidopsis thaliana*	O82383.1
AtUGT71D2	*Arabidopsis thaliana*	O82385.1
BvUGT71B11	*Barbarea vulgaris*	AVW82170.1
BvUGT71B10	*Barbarea vulgaris*	AVW82187.1
BvUGT71B9	*Barbarea vulgaris*	AVW82173.1
BvUGT71C8	*Barbarea vulgaris*	AVW82176.1
BvUGT71D3	*Barbarea vulgaris*	AVW82167.1
MtUGT71G1	*Medicago truncatula*	AAW56092.1
CaUGT71K2	*Cucurbita argyrosperma*	KAG7037474.1
UGT72		
AtUGT72B1	*Arabidopsis thaliana*	Q9M156.1
AtUGT72C1	*Arabidopsis thaliana*	O23205.3
AtUGT72D1	*Arabidopsis thaliana*	Q9ZU72.1
AtUGT72E1	*Arabidopsis thaliana*	OAP04815.1
BvUGT72D3	*Barbarea vulgaris*	AVW82171.1
LjUGT72AF1	*Lotus japonicus*	AOG18239.1
LjUGT72V3	*Lotus japonicus*	AOG18244.1
UGT73		
AtUGT73C5	*Arabidopsis thaliana*	OAP09184.1
AtUGT73C6	*Arabidopsis thaliana*	OAP07438.1
AtUGT73C3	*Arabidopsis thaliana*	Q9ZQ96.1
AtUGT73C4	*Arabidopsis thaliana*	Q9ZQ97.1
AtUGT73C2	*Arabidopsis thaliana*	OAP09975.1
AtUGT73C1	*Arabidopsis thaliana*	OAP11697.1
AtUGT73C7	*Arabidopsis thaliana*	OAP05301.1
AtUGT73D1	*Arabidopsis thaliana*	OAP05971.1
AtUGT73B2	*Arabidopsis thaliana*	OAO97622.1

AtUGT73B3	*Arabidopsis thaliana*	OAO99384.1
AtUGT73B4	*Arabidopsis thaliana*	OAP08080.1
BvUGT73C10	*Barbarea vulgaris*	AFN26666.1
BvUGT73C11	*Barbarea vulgaris*	AFN26667.1
BvUGT73A4	*Barbarea vulgaris*	AAS94329.1
MtUGT73C1	*Medicago truncatula*	XP_003625716.1
AeUGT73AD2	*Aralia elata*	UMX47351.1
CfUGT73C2	*Cornus florida*	XP_059646802.1
LfUGT73C3	*Lycium ferocissimum*	XP_059306976.1
ItUGT73CD1	*Iris tectorum*	WBA33390.1
GeUGT73F1	*Glycyrrhiza echinata*	BAC78438.1
GmUGT73F4	*Glycine max*	BAM29363.1
CtUGTUGT73AE1	*Carthamus tinctorius*	AJT58578.1
GuUGT73F17	*Glycyrrhiza uralensis*	AXS75258.1
UGT74		
AtUGT74F2	*Arabidopsis thaliana*	OAP07463.1
AtUGT74F1	*Arabidopsis thaliana*	OAP07994.1
AtUGT74B1	*Arabidopsis thaliana*	NP_173820.1
AtUGT74E2	*Arabidopsis thaliana*	OAP17332.1
AtUGT74E1	*Arabidopsis thaliana*	P0C7P7.1
AtUGT74D1	*Arabidopsis thaliana*	OAP11252.1
AtUGT74C1	*Arabidopsis thaliana*	Q9SKC1.1
BvUGT74F5	*Barbarea vulgaris*	AVW82180.1
BvUGT74F3	*Barbarea vulgaris*	AVW82183.1
BvUGT74F4	*Barbarea vulgaris*	AVW82182.1
AeUGT74AG6	*Aralia elata*	UMX47352.1
CaUGT74AG2	*Centella asiatica*	AUR26630.1
DcUGT74E2	*Daucus carota*	XP_017242607.1
SgUGT74AC1	*Siraitia grosvenorii*	K7NBW3.1
PgUGT74AE2	*Panax ginseng*	QEA68969.1
PgUGT74A1	*Panax ginseng*	A0A0A6ZFR4.1
UGT75		
AtUGT75C1	*Arabidopsis thaliana*	Q0WW21.2
AtUGT75B2	*Arabidopsis thaliana*	Q9ZVY5.1
AtUGT75B1	*Arabidopsis thaliana*	Q9LR44.1
CsUGT75L12	*Camellia sinensis*	ALO19892.1
AkUGT75L21	*Angelica keiskei*	AWU66065.1
AkUGT75W2	*Angelica keiskei*	AWU66066.1
RcUGT75L20	*Rubus chingii*	AWU66062.1
RcUGT75T4	*Rubus chingii*	AWU66063.1
AsUGT75K6	*Avena strigosa*	AZQ26927.1

HvUGT75E2	*Hordeum vulgare*	KAE8807698.1
AcUGT75D1	*Acorus calamus*	KAK1294935.1
UGT76		
AtUGT76E12	*Arabidopsis thaliana*	Q94AB5.1
AtUGT76E11	*Arabidopsis thaliana*	OAP05192.1
AtUGT76B1	*Arabidopsis thaliana*	OAP05179.1
SrUGT76G1	*Stevia rebaudiana*	AGL95113.1
UGT78		
AtUGT78D3	*Arabidopsis thaliana*	OAO94865.1
AtUGT78D2	*Arabidopsis thaliana*	Q9LFJ8.1
AtUGT78D1	*Arabidopsis thaliana*	OAP13716.1
MtUGT78G1	*Medicago truncatula*	A6XNC6.1
GMUGT78K1	*Glycine max*	NP_001304377.2
UGT79		
AtUGT79B7	*Arabidopsis thaliana*	Q9M0P3.1
AtUGT79B11	*Arabidopsis thaliana*	Q9XIQ4.1
AtUGT79B2	*Arabidopsis thaliana*	Q9T080.1
AtUGT79B1	*Arabidopsis thaliana*	OAO90958.
GMUGT79B3	*Glycine max*	KAH1247062.1
UGT82		
AtUGT82A1	*Arabidopsis thaliana*	Q9LHJ2.1
BrUGT82A1	*Brassica rapa*	XP_009110882.1
UGT83		
AcUGT83A1	*Acorus calamus*	KAK1317019.1
DcUGT83A1	*Dendrobium catenatum*	XP_020671824.1
AsUGT83A1	*Apostasia shenzhenica*	PKA55929.1
UGT84		
AtUGT84A4	*Arabidopsis thaliana*	OAO98847.1
AtUGT84A3	*Arabidopsis thaliana*	OAP00592.1
AtUGT84A2	*Arabidopsis thaliana*	OAP06181.1
AtUGT84A1	*Arabidopsis thaliana*	OAO99238.1
AtUGT84B2	*Arabidopsis thaliana*	OAP08290.1
AtUGT84B1	*Arabidopsis thaliana*	OAP11221.1
UGT85		
CsUGT85C2	*Centaurea solstitialis*	Q6VAB0.1
CsUGT85C1	*Centaurea solstitialis*	Q6VAA4.1
AtUGT85A5	*Arabidopsis thaliana*	OAP19389.1
AtUGT85A4	*Arabidopsis thaliana*	OAP13096.1
AtUGT85A3	*Arabidopsis thaliana*	OAP16353.1
AtUGT85A2	*Arabidopsis thaliana*	OAP18465.1
AtUGT85A1	*Arabidopsis thaliana*	OAP13723.1

UGT87		
AtUGT87A2	*Arabidopsis thaliana*	OAP10737.1
AtUGT87A1	*Arabidopsis thaliana*	O64732.1
UGT89		
AtUGT89C1	*Arabidopsis thaliana*	Q9LNE6.1
AtUGT89B1	*Arabidopsis thaliana*	OAP14423.1
CaUGT89A2	*Cucurbita argyrosperma*	KAG7032141.1
UGT90		
AtUGT90A1	*Arabidopsis thaliana*	Q9ZVX4.1
AtUGT90A2	*Arabidopsis thaliana*	Q9SY84.1
AaUGT90A7	*Artemisia annua*	PWA57943.1
UGT91		
AtUGT91A1	*Arabidopsis thaliana*	Q940V3.1
BvUGT91A10	*Barbarea vulgaris*	AVW82166.1
AtUGT91B1	*Arabidopsis thaliana*	Q9LSM0.1
AtUGT91C1	*Arabidopsis thaliana*	Q9LTA3.1
GmUGT91H9	*Glycine max*	NP_001348424.1
GmUGT91H4	*Glycine max*	NP_001240857.1
CaUGT91D2	*Cucurbita argyrosperma*	KAG6582627.1
UGT92		
AtUGT92A1	*Arabidopsis thaliana*	Q9LXV0.1
UGT94		
PgUGT94G2	*Panax ginseng*	A0A0A6ZFY4.1
UGT95		
PoUGT95A1	*Pilosella officinarum*	ACB56927.1
FtUGT95GD1	*Fagopyrum tataricum*	QAV53742.1
FtUGT95GD1	*Fagopyrum tataricum*	QAV53742.1
